# Italian Registry of Congenital Bleeding Disorders

**DOI:** 10.3390/jcm6030034

**Published:** 2017-03-19

**Authors:** Adele Giampaolo, Francesca Abbonizio, Romano Arcieri, Hamisa Jane Hassan

**Affiliations:** 1 Department of Oncology and Molecular Medicine, Istituto Superiore di Sanità, 00161 Rome, Italy; francesca.abbonizio@iss.it (F.A.); jane.hassan@iss.it (H.J.H.); 2 Grant Office and Technology Transfer, Istituto Superiore di Sanità, 00161 Rome, Italy; romano.arcieri@iss.it

**Keywords:** registry, bleeding disorders, Hemophilia A, Hemophilia B, rare diseases, clotting factors

## Abstract

In Italy, the surveillance of people with bleeding disorders is based on the National Registry of Congenital Coagulopathies (NRCC) managed by the Italian National Institute of Health (Istituto Superiore di Sanità). The NRCC collects epidemiological and therapeutic data from the 54 Hemophilia Treatment Centers, members of the Italian Association of Hemophilia Centres (AICE). The number of people identified with bleeding disorders has increased over the years, with the number rising from approx. 7000 in 2000 to over 11,000 in 2015. The NRCC includes 4020 patients with hemophilia A and 859 patients with hemophilia B. The prevalence of the rare type 3 vWD is 0.20/100,000 inhabitants. Less common congenital bleeding disorders include the following deficiencies: Factor I (fibrinogen), Factor II (prothrombin), Factor V, Factor VII, Factor X, Factor XI and Factor XIII, which affect 1953 patients. Hepatitis C Virus (HCV) infection affects 1561 patients, more than 200 of whom have two infections (HCV + HIV). Estimated hemophilia-related drug consumption in 2015 was approx. 550 million IU of FVIII for hemophilia A patients and approx. 70 million IU of FIX for hemophilia B patients. The NRCC, with its bleeding disorder data set, is a tool that can provide answers to fundamental questions in public health, monitoring care provision and drug treatment, as well as facilitating clinical and epidemiological research.

## 1. Introduction

Blood coagulation is a complex mechanism that is required for the rapid establishment of a stable fibrin clot. A series of interdependent enzyme-mediated reactions translate the molecular signals that initiate blood coagulation into the formation of the fibrin clot. Congenital coagulopathies result when there is a deficiency of protein cofactors and enzymes implicated in blood coagulation [1].

The most frequent coagulopathies are Hemophilia A (HA, factor VIII deficiency), Hemophilia B (HB, factor IX deficiency) and von Willebrand Disease (vWD) [2]. Less common congenital bleeding disorders include deficiencies of Factor I (fibrinogen), Factor II (prothrombin), Factor V, Factor VII, Factor X, Factor XI and Factor XIII [1]. Therapy consists of replacing the deficient clotting factor through the administration of the specific factor concentrate; when the specific factor concentrates are not available, multi-factor concentrates, such as prothrombin complex concentrates, or human plasma (fresh-frozen plasma or commercially available products) can be used.

The World Federation of Hemophilia and the European Hemophilia Consortium have long highlighted the need for the institution and implementation of national specific registries dedicated to hemophilia and congenital coagulopathies [3,4].

The first such registry was the United Kingdom National Hemophilia Database, which was set up in 1969 and is managed by the United Kingdom Hemophilia Centre Doctors’ Organization (UKHCDO). The UKHCDO is required by the National Department of Health to collect data, through a web-based system, on the diagnosis, management and complications of bleeding disorders [5].

The Canadian Hemophilia Registry (CHR) was set up in 1988 to enumerate the number of individuals with hemophilia A and B, and now continues under the Association of Hemophilia Clinic Directors of Canada with the inclusion of a ‘von Willebrand’ registry and the Rare Inherited Bleeding Disorders Registry [6]. 

In Italy, the surveillance of people with bleeding disorders is based on the National Registry of Congenital Coagulopathies (NRCC) managed by the Italian National Institute of Health (Istituto Superiore di Sanità). Between 1988 and 1999, this institute provided reports aimed, above all, at the surveillance of HIV infection, which seriously affected the hemophiliac population at that time [7,8]. A new collection system has been set up since then together with the Italian Association of Hemophilia Centres (AICE), which has led to data on bleeding disorders being collected and published annually since 2005 [9,10]. The NRCC has been included in the National Statistics System (SISTAN), which conducts surveys of public interest (www.sistan.it).

In this paper, we provide epidemiological information on patients with bleeding disorders as well as an estimate of FVIII and FIX products required in Italy to treat HA and HB patients using data collected in 2015 by the Italian registry of bleeding disorders. 

## 2. Materials and Methods

The NRCC annually collects data from the 54 Hemophilia Treatment Centers (HTCs) in the country, which are distributed in the north (49%), centre (17%) and south and islands (34%) of Italy. The subjects included in the registry are patients receiving care in the HTCs who have previously been asked to sign a consent form to allow data collection for epidemiological and clinical research purposes. The patients’ demographic and clinical data are recorded in a web-based platform, developed by the Italian Association of Hemophilia Centers (AICE), and shared anonymously with the National Institute of Health, fully respecting the patients’ privacy, confidentiality and security and in accordance with Italian privacy law and standards. In the NRCC, patients are classified by the clinicians in the HTCs according to their diagnosis, severity, age, gender and treatment-related complications, such as infections and alloantibodies to FVIII or FIX. The population included in the registry is affected by HA, HB, vWD, other congenital factor defects or congenital platelet disorders; data on HA and HB carriers are also provided.

A specific section of the registry is dedicated to the drugs required to treat the patients. Data are collected on the basis of the therapeutic plans, which are mandatory by law and indicate the diagnosis of the bleeding disorder, its severity, treatment regimen, time covered by the therapeutic plan (from 1 month up to 1 year, on the basis of a personalized clinical approach), dosage, source (plasma-derived or recombinant) and brand name of the product, and the total number of International Units (IU) assigned. The data regarding the use of products is normalized to each patient’s annual consumption.

Data on patients’ prescribed annual product consumption of severe hemophilia patients recorded in the registry are based on 75% of this population. We estimated the consumption for the entire severe hemophilia population in Italy by assuming that the percentage of subjects undergoing prophylaxis and on-demand treatment among the missing 25% of patients is the same as that of the patients for whom we collected data. For each regimen, the median FVIII/FIX consumption was multiplied by the number of missing patients and added to the value yielded by the 75% of patients for whom data were available.

## 3. Results

**Figure 1:**
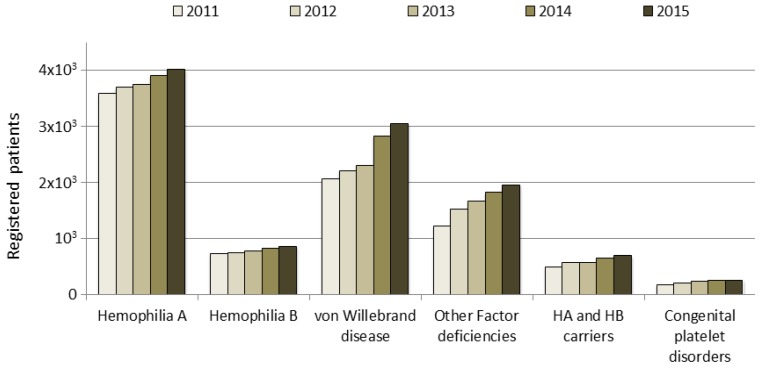
Trend in the total number of patients with bleeding disorders registered in the National Registry of Congenital Coagulopathies (NRCC) from 2011 to 2015. HA: Hemophilia A; HB: Hemophilia B.

In Italy, the number of registered people with bleeding disorders increased from approximately 7000 in 2000 to 8500 in 2011 and to over 11,000 in 2015; this trend is due to an increase in the number of patients who are registered, particularly in those with vWD type 1, mild hemophilia or other factor deficiencies. These data do not include deceased people, whose data are analyzed in a separate section of the NRCC (data not shown). The increase in the number of patients registered according to clotting factor deficiency in the last five years is shown in Figure 1. Patients are monitored by their HTCs through an annual check-up, as recommended by AICE, and their data updated in the registry.

**Figure 2:**
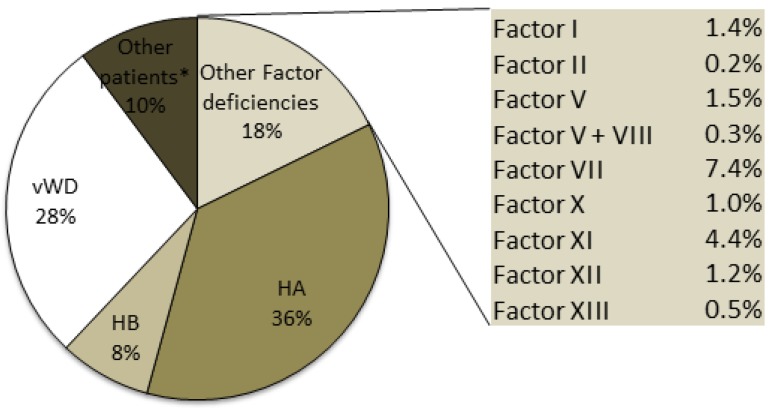
Patients with inherited bleeding disorders recorded in the NRCC-2015. * “Other patients” include HA and HB carriers and patients with congenital platelet disorders. vWD: von Willebrand Disease.

Hemophilia A, which is the most frequent disorder, is transmitted as an X-linked disorder that affects male subjects: 4020 HA patients were identified, accounting for 36% of the total number of patients recorded in 2015 (Figure 2).

The severe form affected 46% of the total number of patients with HA, including three women. Patients in the under 18-years of age group with severe, moderate and mild HA accounted for 22.1%, 19.4% and 12.9% of the overall number of patients with each of these forms of HA (Table 1). The higher proportion of patients with the severe and moderate forms than of patients with the mild form points to a delay in the diagnosis in the mild form group.

Individuals with HB (859) account for 8% of the total number of patients recorded, with 36% of these HB patients having the severe form. The data collected for the under 18-years of age group with HB revealed that the patients in this group with the severe, moderate and mild forms accounted for 25.1%, 18.5% and 19.5% respectively of the overall number of patients with each of these forms of HB (Table 1).

Although vWD is described as the most common hereditary coagulation disorder, only 3047 patients with vWD were included in the registry (28% of the total number of patients): 76% of these vWD patients were affected by type 1, the most common form, which is characterized by quantitative defects and an autosomal dominant inheritance. The rare type 3 form, which is characterized by a more severe quantitative deficiency and is transmitted as an autosomal recessive form, accounted for 4% of the total number of patients with vWD; the other patients with vWD included in the registry were affected by the four different type 2 forms. Patients in the under 18-years of age group with the vWD type 1 and 2 forms accounted for about 10% of the overall number of patients with these two forms, while those with the vWD type 3 form accounted for 8.4% of the overall number of patients with this form (Table 1).

On the basis of data collected by the National Institute of Statistics in 2015 for the Italian male population (29,456,321), the prevalence of the two forms of hemophilia is 1.36/10,000 males for HA and 0.29/10,000 males for HB. The prevalence of the rare type 3 vWD was 0.20/100,000 inhabitants.

The other rare inherited bleeding disorders reported in the registry (Figure 2) are deficiencies of Factors I, II, V, VII, X, XI, XII, XIII and combined V+VIII, which are usually transmitted as autosomal recessive disorders and affect 1953 patients. The most frequent deficiency of the rare bleeding disorders was FVII, with more than 800 recorded cases and a prevalence in the Italian population of 1.32/100,000 inhabitants. The least frequent deficiency was prothrombin deficiency, with 20 recorded cases that were equally distributed between male and female subjects.

The drug-related adverse events for which patients are monitored include the presence of HIV and Hepatitis C Virus (HCV) infections and of alloantibodies to FVIII and FIX (Table 2).

Two hundred and fifty-three patients with HIV infection were recorded in 2015; the last case of a patient being infected with HIV was recorded 30 years ago. Patients with severe HA accounted for 65.6% of the infected HIV population. HCV infection affected 1561 patients, more than 200 of whom had two infections (HCV + HIV).

Alloantibodies to FVIII and FIX were detected in 8.1% of Italian hemophilic patients in 2015, and in approximately 30% of the youngest patients (0–3 years). In particular, in the severe HA population (1838 patients), a history of alloantibodies to FVIII was recorded in 18.8%, while patients with alloantibodies to FVIII being treated with by-passant agents, to control bleeding, or Immune Tolerance Induction, to attempt inhibitor eradication and restore FVIII therapy, accounted for 6% of the population.

**Figure 3:**
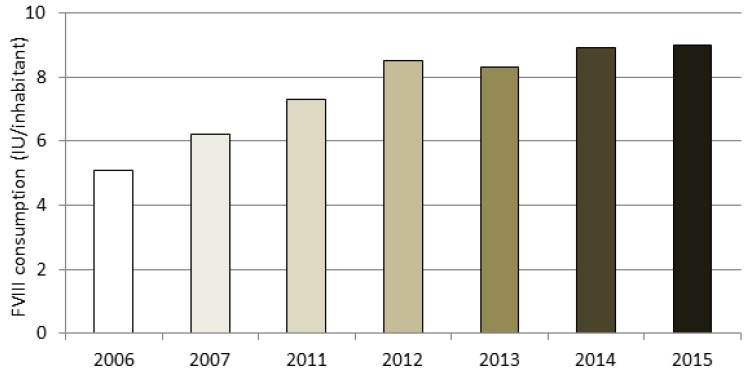
Trend of estimated FVIII used in Italy over the years based on the entire population of severe HA.

A section of the NRCC is dedicated to data on HA and HB drug therapy. The amount of plasma-derived and recombinant FVIII used in Italy is estimated by analyzing the mandatory therapeutic plans prescribed by clinicians to patients. The data are based on 75% of the severe hemophiliacs being treated. By assuming that the percentage of subjects undergoing prophylaxis and on-demand treatment among the missing 25% of patients is the same as that of the patients for whom we collected data (as described in “Methods”), the estimated FVIII consumption for the entire severe HA population was about 550 million IU, which corresponds to 9.0 IU per capita. It is reasonable to assume that the increasing trend in IU consumption per capita (shown in Figure 3) is due to an increased use of secondary prophylaxis, according to AICE Recommendations, as well as to an increase in tertiary prophylaxis.

Eighty-four percent of the total amount of FVIII was assigned to the treatment of severe HA, 8% to that of moderate HA and 4% to that of mild HA patients, while the remaining 4% was assigned to the treatment of patients with vWD.

The use of plasma-derived FVIII, which accounted for 23% of the total amount, has increased in recent years (19% in 2011 and 21% in 2013). The distribution of recombinant FVIII in the different age groups indicates that almost all the youngest patients (<10 years) have used recombinant products, and that consumption decreases with age, dropping to approximately 55% in the 50–60-years of age group.

Prophylaxis is the therapeutic regimen chosen by 72% of patients with severe HA in the 0–20-years of age group, by 57% in the 20–40-years of age group and by approximately 43% in the over 40-years of age group. A different ratio was observed between prophylaxis and on-demand treatment in patients with HB, with prophylaxis being chosen by 55% of severe HB patients and 80% of patients in the 0–20-years of age group.

The estimated FIX consumption was about 70 million IU for all the HB patients, which corresponds to 1.2 IU per capita, 83% of which was used in the recombinant form.

## 4. Discussion

Blood coagulation disorders are rare diseases that include hemophilia A and B, vWD and other clotting factor deficiencies and that are followed at hemophilia treatment centers. The Italian prevalence of HA, compared with that of UK and Canada—estimated on the basis of the Report on the World Federation of Hemophilia (WFH) Annual Global Survey 2015—is 1.36 vs. 1.96 and 1.79 respectively, while that of HB is 0.29 vs. 0.45 and 0.41. The Italian prevalence falls within the range reported in more developed countries [11,12].

The 54 HTCs in Italy collect data and are invited to participate in the NRCC [9,10]. They assist Italian patients with bleeding disorders, providing treatment for almost 100% of the patients with severe forms; this is due to the fact that patients with severe deficiencies need to receive therapy frequently and a prescription from HTCs is required by law to receive such therapy free of charge. Patients with mild forms, who do not bleed spontaneously frequently, may be diagnosed later (e.g., after major surgery or trauma), which means their referral to HTCs may also be delayed.

The importance of monitoring patients by means of a disease registry has been clearly stated by organizations such as the World Federation of Hemophilia, the European Consortium and the European Commission for rare diseases [13,14]. In Italy, surveillance of patients with bleeding disorders has been managed by the Istituto Superiore di Sanità since 1988. Over the years, the scope of the Italian registry has changed from the surveillance of viral infections in the hemophiliac population to the current NRCC, which has a range of objectives: the epidemiology of blood clotting disorders, the surveillance of patients through information on adverse events and comorbidities, and drug treatment. The NRCC is the result of the collaboration between a number of stakeholders: government institutions, medical associations (AICE) and patient associations. Data are available for different purposes: epidemiological studies, clinical studies and treatment planning and appropriateness.

The life expectancy of patients with bleeding disorders has become comparable to that of the general population [15]. Risk factors for viral infection are, following increased safety of the drug products, now similar to those to which the general population is exposed. Indeed, the last recording of a patient with a bleeding disorder who contracted HIV infection occurred 30 years ago. However, almost 1600 patients are HCV infected and hepatocellular carcinoma accounts for 50% of the tumors in this population (15 and our unpublished results). In this regard, the strict surveillance of patients with bleeding disorders should be maintained in view of the potential exposure to new infectious agents as well as to new products. At present, the main adverse event to therapy in hemophilia patients is the development of alloantibodies to FVIII and FIX, which affects as many as 30% of the 0- to 3-year-old population of HA patients. Given its negative impact on the quality of life of patients and their families, and the economic repercussions on the national health system, research is being conducted to gain an understanding of the possible causes and to attempt to eradicate alloantibodies. Numerous risk factors are being investigated in relation to the development of alloantibodies: genetic, age, type of product used and intensity of exposure to the treatment regimen [16,17,18,19]. The distribution of recombinant FVIII in the different age groups indicates that almost all the youngest patients (<10 years) have used recombinant products. As this subject is currently strongly debated, particularly as regards the impact of the product [16], the orientation of specialists and the perception of patient associations need to be closely monitored. The registry collects any trends or changes in product usage and monitors the development of alloantibodies. Data collected by the registry on FVIII and FIX consumption in Italy, regardless of the product used, yielded values of 9.0 IU per capita and 1.2 IU per capita, respectively; these figures for the use of prophylaxis treatment are in keeping with data collected in other European countries [20]. The increasing trend in FVIII consumption per capita, shown in Figure 3, is reasonable due to an increased use of secondary prophylaxis, according to AICE Recommendations, as well as to an increase in tertiary prophylaxis.

A national bleeding disorders registry containing a data set that is specific to different disease groups facilitates clinical and epidemiological research that can subsequently be accessed by researchers and policy makers. A national registry may also be used to identify critical issues on a regional scale so as to be able to plan and implement corrective initiatives [21].

Although the NRCC was set up many years ago, there is room for improvement as regards the data collected and the completeness of case notifications. The quality of the data collected has been improved over the years through standardized controls (i.e., presence of duplicates, correctness of the dates of birth, diagnosis, verification of the status in life and matching between the diagnosis and therapy assigned). This system has led to requests being made for missing data and to non-matching data being reported to the HTCs for verification and correction.

The introduction of new drugs, such as extended half-life (EHL) Factor VIII and Factor IX products [22], represents a new challenge in public health that requires post-marketing surveillance that the NRCC, as a disease-specific registry, can support.

## Figures and Tables

**Table 1:** Distribution of patients with HA, HB and vWD, according to disease severity.

Pathology	Total Patients	<18 Years Old	Median Age (0–90 Years Old)
Severe HA	1838	406 (22.1%)	35
Moderate HA	552	107 (19.4%)	37
Mild HA	1630	210 (12.9%)	42
**Hemophilia A**	**4020**	**723 (18.0%)**	**38**
Severe HB	311	78 (25.1%)	32
Moderate HB	184	34 (18.5%)	38
Mild HB	364	71 (19.5%)	38
**Hemophilia B**	**859**	**183 (21.3%)**	**36**
vWD type 1	2307	248 (10.8%)	42
vWD type 2	621	66 (10.6%)	46
vWD type 3	119	10 (8.4%)	41
**Von Willebrand Disease**	**3047**	**324 (10.6%)**	**43**

**Table 2:** Patients with major adverse events (presence of HIV and Hepatitis C Virus (HCV) infection and of alloantibodies to FVIII and FIX) recorded in the NRCC-2015.

Pathology	HIV Infection	HCV Infection	Alloantibodies
Severe HA	166	743	345
Moderate HA	13	178	20
Mild HA	10	293	15
Severe HB	40	101	13
Moderate HB	10	44	-
Mild HB	2	37	-
vWD type 1	4	54	-
vWD type 2	2	30	-
vWD type 3	3	23	-
Factor I	-	4	-
Factor II	-	1	-
Factor V	-	3	-
Factor V + FVIII	-	6	-
Factor VII	3	21	-
Factor X	-	1	-
Factor XI	-	16	-
Factor XII	-	1	-
Factor XIII	-	2	-
Not indicated	-	3	-
**Total**	**253**	**1561**	**393**
